# Nuclear factor of activated T cells 4 in the prefrontal cortex is required for prophylactic actions of (*R*)-ketamine

**DOI:** 10.1038/s41398-022-01803-6

**Published:** 2022-01-21

**Authors:** Li Ma, Jiancheng Zhang, Yuko Fujita, Youge Qu, Jiajing Shan, Xiayun Wan, Xingming Wang, Tamaki Ishima, Kenta Kobayashi, Long Wang, Kenji Hashimoto

**Affiliations:** 1 Division of Clinical Neuroscience, Chiba University Center for Forensic Mental Health, Chiba, 260-8670 Japan; 2 Department of Anesthesiology, Renmin Hospital of Wuhan University, Wuhan, 430060 Hubei Province China; 3 Section of Viral Vector Development, Center for Genetic Analysis of Behavior, National Institute for Physiological Sciences, Okazaki, Aichi 444-8585 Japan; 4 Present Address: Department of Critical Care Medicine, Union Hospital, Tongji Medical College, Huazhong University of Science and Technology, Wuhan, 430022 China

**Keywords:** Pharmacogenetics, Depression

## Abstract

(*R, S*)-ketamine has prophylactic antidepressant-like effects in rodents; however, the precise molecular mechanisms underlying its action remain unknown. Using RNA-sequencing analysis, we searched novel molecular target(s) that contribute to the prophylactic effects of (*R*)-ketamine, a more potent enantiomer of (*R, S*)-ketamine. Pretreatment with (*R*)-ketamine (10 mg/kg, 6 days before) significantly ameliorated body weight loss, splenomegaly, and increased immobility time of forced swimming test in lipopolysaccharide (LPS: 1.0 mg/kg)-treated mice. RNA-sequencing analysis of prefrontal cortex (PFC) and subsequent IPA (Ingenuity Pathway Analysis) revealed that the nuclear factor of activated T cells 4 (NFATc4) signaling might contribute to sustained prophylactic effects of (*R*)-ketamine. Quantitative RT-PCR confirmed that (*R*)-ketamine significantly attenuated the increased gene expression of NFATc4 signaling (*Nfatc4, Cd4, Cd79b, H2-ab1*, *H2-aa*) in the PFC of LPS-treated mice. Furthermore, pretreatment with NFAT inhibitors (i.e., NFAT inhibitor and cyclosporin A) showed prophylactic effects in the LPS-treated mice. Similar to (*R*)-ketamine, gene knockdown of *Nfatc4* gene by bilateral injection of adeno-associated virus (AAV) into the mPFC could elicit prophylactic effects in the LPS-treated mice. In conclusion, our data implicate a novel NFATc4 signaling pathway in the PFC underlying the prophylactic effects of (*R*)-ketamine for inflammation-related depression.

## Introduction

Robust antidepressant action of the *N*-methyl-D-aspartate receptor (NMDAR) antagonist (*R, S*)-ketamine is a paradigm shift for depression research and treatment [1]. In 2000, Berman et al. [2] demonstrated the rapid-onset and sustained antidepressant actions of (*R, S*)-ketamine in patients with major depressive disorder (MDD). Subsequently, several groups replicated the robust antidepressant effects of (*R, S*)-ketamine in treatment-resistant patients with MDD or bipolar disorder (BD) [3–10]. Meta-analyses revealed that (*R, S*)-ketamine has rapid-acting and sustained antidepressant effects in treatment-resistant patients with MDD or BD [11–13]. Although (*R, S*)-ketamine can produce the robust antidepressant actions in severe patients with depression, precise molecular mechanisms underlying its antidepressants remain elusive [14–22].

Dr. Denny and her colleagues demonstrated that (*R, S*)-ketamine could produce persistent prophylactic effects against chronic social defeat stress (CSDS) model, learned helplessness (LH) model, chronic corticosterone-treated model [23], and lipopolysaccharide (LPS)-treated inflammation model [24]. Furthermore, the same group reported prophylactic effects of (*R, S*)-ketamine against fear expression [25, 26]. It is also reported that ΔFosB in the ventral CA3 of hippocampus plays a role in the prophylactic effects of (*R, S*)-ketamine in CSDS model [27]. Moreover, it is demonstrated that (*R, S*)-ketamine produced a robust pro-resilient response to CSDS through Akt signaling in the ventral tegmental area (VTA)-nucleus accumbens (NAc) [28]. Interestingly, Ma et al. [29] reported prophylactic effects of (*R, S*)-ketamine on postpartum depression in Chinese women undergoing cesarean section. Collectively, it is possible that (*R, S*)-ketamine may be useful in protecting against stress-related psychiatric disorders such as depression and posttraumatic stress disorder (PTSD) [30]. However, the precise molecular and cellular mechanisms underlying prophylactic actions of (*R, S*)-ketamine remain unclear.

(*R,S*)-ketamine (Ki = 0.53 μM for NMDAR) is a racemic mixture that contains equal amounts of (*R*)-ketamine (or arketamine) (Ki = 1.4 μM for NMDAR) and (*S*)-ketamine (or esketamine) (Ki = 0.30 μM for NMDAR). In 2019, (*S*)-ketamine nasal spray for treatment-resistant MDD patients was approved in the United State and Europe. In contrast, increasing preclinical data show that (*R*)-ketamine displays greater potency and longer-lasting antidepressant effects than (*S*)-ketamine in rodent models of depression [31–41], suggesting that NMDAR does not play a major role in the robust antidepressant-like effects of (*R, S*)-ketamine. Importantly, side effects of (*R*)-ketamine are less than those of (*R, S*)-ketamine or (*S*)-ketamine [32, 38, 42–46]. A recent pilot study demonstrated that (*R*)-ketamine elicited rapid-acting and sustained antidepressant actions in treatment-resistant MDD patients, and that side effects such as dissociation were very low [47]. Taken all together, it is likely that (*R*)-ketamine would be a novel antidepressant without side effects of (*R, S*)-ketamine [16–20, 22]. Meanwhile, there are no articles reporting the prophylactic effects of (*R*)-ketamine in rodents. Little is known about the precise molecular mechanisms underlying the prophylactic effects of (*R*)-ketamine.

The aim of this study was to identify the novel molecular mechanisms underlying the prophylactic effects of (*R*)-ketamine in LPS-induced inflammation model. First, we conducted RNA-sequencing analysis of the prefrontal cortex (PFC) of LPS-treated mice treated with either (*R*)-ketamine or 0.9% saline, as PFC contributes to the antidepressant-like actions of ketamine and its enantiomers [32, 48, 49]. Furthermore, we examined the prophylactic effects of (*R*)-ketamine on LPS-induced splenomegaly in mice since LPS increased spleen weight through systemic inflammation [50]. Second, we studied the effects of pharmacological inhibitors and adeno-associated virus (AAV) vector of the novel target in the prophylactic effects of (*R*)-ketamine in LPS-treated mice.

## Methods and Materials

### Animals

Male adult C57BL/6 mice (8 weeks old, body weight 20–25 g) were purchased from Japan SLC, Inc. (Hamamatsu, Shizuoka, Japan). Animals were housed under controlled temperature and 12 h light/dark cycles (lights on between 07:00–19:00), with *ad libitum* food and water. The study was approved by the Chiba University Institutional Animal Care and Use Committee (1-374, 2-146, and 3-282). All efforts were made to minimize suffering. The sample size was chosen as reported previously.

### Compounds and treatment

(*R*)-ketamine hydrochloride was prepared by recrystallization of (*R, S*)-ketamine (Ketalar^®^, ketamine hydrochloride, Daiichi Sankyo Pharmaceutical Ltd., Tokyo, Japan) and D-(-)-tartaric acid, as reported previously [31]. (*R*)-norketamine hydrochloride was synthesized as reported previously [33]. (2*R*,6*R*)-hydroxynorketamine (HNK) hydrochloride was purchased from Tocris Bioscience (Tokyo, Japan). The dose (10 mg/kg as hydrochloride salt) of (*R*)-ketamine, (*R*)-norketamine, and (2*R*,6*R*)-HNK were selected as reported previously [32, 35–37, 51]. LPS (L-4130, serotype 0111:B4, Sigma-Aldrich, St Louis, MO, USA) was dissolved in saline. The dose (1.0 mg/kg) of LPS was used as reported previously [24]. The NFAT inhibitor (L-methionyl-L-alanylglycyl-L-prolyl-L-histidyl-L-prolyl-L-valyl-L-isoleucyl-L-valyl-L-isoleucyl-L-threonylglycyl-L-prolyl-L-histidyl-L-α-glutamyl-L-glutamic acid, Cat No.: 249537-73-3, Cayman Chemical, Ann Arbor, Michigan, USA) or cyclosporin A (CysA; Cat No.: 59865-13-3, FUJIFILM, Tokyo, Japan) was dissolved in 10% dimethylsulfoxide (DMSO). The dose (40 mg/kg) of CysA was used as reported previously [52]. ANA-12 (0.5 mg/kg; Maybridge, Cornwall, UK), was dissolved in phosphate-buffered saline (PBS) containing 17% DMSO and administrated intraperitoneally (i.p.) to mice 30 min prior to the administration of saline or (*R*)-ketamine, as reported previously [32, 53–57].

### LPS-induced depression model, and behavioral tests

The mice were randomly divided into the groups. The procedure of LPS-treated inflammation model for depression was performed as reported previously [35, 50, 57, 58]. Locomotion test and forced swimming test (FST) were performed 23 and 24 h after i.p. administration of saline (10 ml/kg) or LPS (1.0 mg/kg), respectively. Behavioral tests were performed in a blind manner. Detailed methods were shown in the supplemental information.

### Collection of blood and spleen

The mice were deeply anesthetized with inhaled isoflurane (5%) 24 h after the i.p. injection of saline (10 ml/kg) or LPS (1.0 mg/kg). Blood was collected via cardiac puncture, placed into tubes containing ethylenediaminetetraacetic acid (EDTA), and immediately centrifuged at 3000 × *g* for 3 min at 4 °C to obtain plasma, and then stored at −80 °C until bioanalysis, as reported previously [50]. Prefrontal cortex (PFC) was collected rapidly and stored at −80 °C until bioanalysis. The weight of spleens was recorded immediately after spleen removal.

### Measurement of pro-inflammatory cytokines in the blood

The plasma levels of interleukin-6 (IL-6) and tumor necrosis factor-α (TNF-α) were determined using ELISA kits (IL-6: cat number: 88-7064, TNF-α: cat number: 88-7324, Invitrogen, Camarillo, CA, USA) according to the manufacturer’s instructions.

### RNA-sequencing analysis

**Fig. 1:**
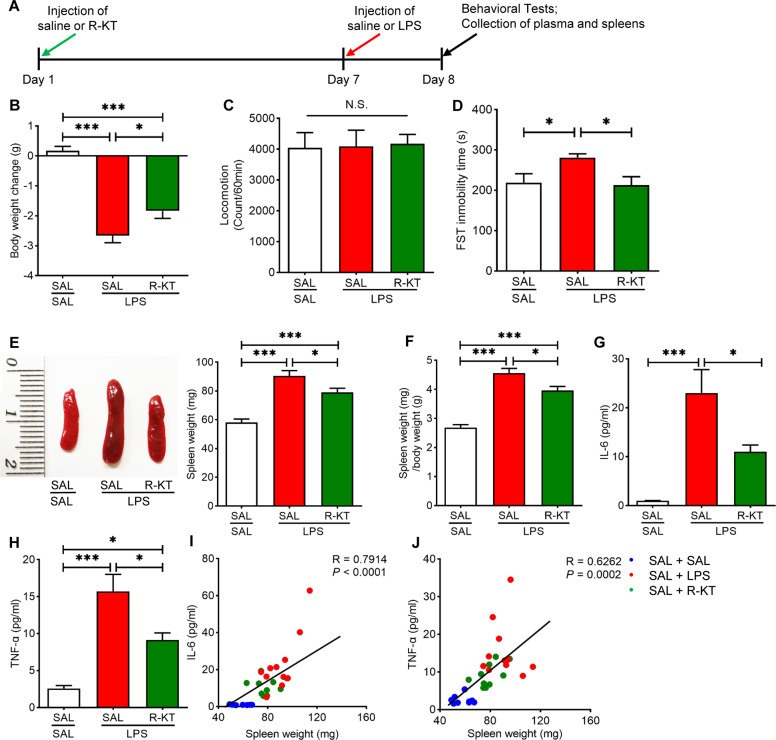
Prophylactic effects of (*R*)-ketamine on depression-like phenotype, splenomegaly and inflammatory cytokines after LPS injection. **A** Treatment schedule. Adult mice were intraperitoneally (i.p.) injected with lipopolysaccharides (LPS, 1.0 mg/kg) or saline (10 ml/kg). (*R*)-ketamine (10 mg/kg) or saline (10 ml/kg) was i.p. injected to mice 6 days before LPS injection. Locomotion test and forced swimming test (FST) were performed 23 and 24 h after the injection of saline or LPS. Blood and spleens were collected after behavioral tests. **B** Body weight change (one way ANOVA: F_2,24_ = 41.19, *P* < 0.0001). **C** Locomotion test (one way ANOVA: F_2,24_ = 0.034, *P* = 0.966). **D** FST (one way ANOVA: F_2,24_ = 4.738, *P* = 0.018). **E** Representative picture of spleen and spleen weight (one way ANOVA: F_2,27_ = 27.06, *P* < 0.001). **F** The ratio of spleen weight/body weight (one way ANOVA: F_2,27_ = 44.13, *P* < 0.0001). **G** Plasma levels of interleukin (IL)-6 (one way ANOVA: F_2,27_ = 12.24, *P* = 0.0002). **H** Plasma levels of tumor necrosis factor (TNF)-α (one way ANOVA: F_2,27_ =17.08, *P* < 0.0001). **I** There was a positive correlation (*R* = 0.791, *P* < 0.0001) between spleen weight and plasma IL-6. (**J**): There was a positive correlation (*R* = 0.626, *P* = 0.0002) between spleen weight and plasma TNF-α. The data represent mean ± S.E.M. (*n* = 8–11). ^*^P < 0.05, ^**^P < 0.01, ^***^P < 0.0001. N.S., not significant.

(*R*)-ketamine (10 mg/kg) or saline (10 ml/kg) was administered i.p. to mice 6 days before i.p. administration of saline (10 ml/kg, i.p.) or LPS (1.0 mg/kg, i.p.) (Fig. 1A). PFC was collected 24 h after a single administration of saline or LPS. RNA-sequencing analysis of the PFC samples was performed at the Novogene (Beijing, China). Analysis of the biological functions was performed using the Ingenuity pathway Analysis (IPA) [59].

### Quantitative real-time PCR

Saline (10 ml/kg) or (*R*)-ketamine (10 mg/kg) was administered i.p. to mice 6 days before LPS (1.0 mg/kg, i.p.) administration. Mice were sacrificed 24 h after administration of saline or LPS. Mice were sacrificed under deep anesthesia by isoflurane (5%), then PFC was quickly dissected on ice from the whole brain.

A quantitative RT-PCR system (Step One Plus, Thermo Fisher Scientific, Yokohama, Japan) was used. All specific mRNA transcripts were quantitatively analyzed by TaqManGene Expression assays (Thermo Fisher Scientific, Yokohama, Japan). The gene expression levels of *Cd4* (Mm00442754_m1), *Cd79b* (Mm00434143_m1), *H2-Aa* (Mm00429211_m1), *H2-Ab1* (Mm00439216_m1), *Nfatc4*(Mm00452375_m1) were measured. Total RNA was extracted using an RNase-Free DNase Set and a RNeasy Mini Kit (Qiagen, Hilden, Germany). The purity of total RNA was assessed by Bio photometer plus (Eppendorf, Hamburg, Germany). The cDNA libraries were obtained by reverse transcription-PCR using a High-Capacity cDNA Reverse Transcription Kit (#4368813 Thermo Fisher Scientific, Yokohama, Japan). All specimens were detected twice, and arithmetic means were used for quantification. The data of arithmetic mean were normalized to Vic-labeled *Actb* mRNA (#4352341E: pre-developed TaqMan Assay Reagents, Thermo Fisher Scientific, Yokohama, Japan).

### Effects of NFAT inhibitors

To examine the role of NFATc4 in the prophylactic effects of (*R*)-ketamine, the two inhibitors (NFAT inhibitor and CysA) of NFATc4 were used. The NFAT inhibitor (10 μM, 2 μl, i.c.v.) or saline (2 μl, i.c.v.) was injected 60 min before i.p. administration of LPS (1.0 mg/kg) in mice. CysA (40 mg/kg, i.p.) or vehicle (10% DMSO, 10 ml/kg, i.p.) was injected 60 min before i.p. administration of LPS (1.0 mg/kg) in mice. Subsequently, the behavioral tests such as locomotion test and FST were performed as described above.

### Viral vector preparation and injection

The transfer plasmid [U6-sh*Nfatc4* (short hairpin RNA against *Nfatc4*)-CAGGS-EmGFP] was constructed by Invitrogen. The viral vectors were prepared as described previously [60]. Briefly, the AAV vectors were packaged using the AAV Helper Free Expression System (Cell Biolabs, Inc., San Diego, CA). The packaging plasmids (pAAV-DJ and pHelper) and transfer plasmid (pAAV-U6- shRNA-CAGGS-EmGFP or pAAV- U6-CAGGS-EGFP) were transfected into HEK293T cells using the calcium phosphate method. After 48 h incubation, AAV vector particles were obtained and purified by serial ultracentrifugation with cesium chloride. The purified particles were dialyzed with PBS containing 0.001% Pluronic F-68 (Sigma-Aldrich, St. Louis, MO), and then concentrated by ultrafiltration using an Amicon 10k MWCO filter (Merck Millipore, Darmstadet, Germany). The copy number of the viral genome (vg) was determined by the TaqMan Universal Master Mix II (Applied Biosystems, Foster City, CA). Real-time quantitative PCR was performed in duplicate samples using the StepOne real-time PCR system as follows: 95 °C for 10 min; 40 cycles of (95 °C, 15 s, and 60 °C, 1 min).

To induce gene expression in the mPFC, AAV DJ-CAGGS-Nfatc4-P2A-EmGFP or AAV DJ-CAGGS-EGFP vectors (1.0 × 10^12^ vg/ml) were bilaterally injected into the mPFC (+1.7 AP, ±0.4 ML, −1.8 DV) of C57BL/6 male mice at 9 weeks old by microinjection tube connected to a micro-infusion pump (1 μl/site, 0.5 μl/min) [61]. Three weeks after injection, saline (10 ml/kg) or LPS (1.0 mg/kg) was administered i.p. to mice. Subsequently, behavioral tests such as locomotion test and FST were performed. After behavioral tests, the bilateral medial prefrontal cortex (mPFC) was collected rapidly and stored at −80°C until bioanalysis. The weight of spleens was recorded immediately after spleen removal.

### Western blot analysis

Detailed methods for Western blot analysis were shown in the supplemental information.

### Statistical analysis

The data were shown as mean ± standard error of the mean (S.E.M.). Analysis was performed using PASW Statistics 20 (formerly SPSS Statistics; SPSS). A test of homogeneity of variance for all animal data showed no significant difference. The data were analyzed using the one-way analysis of variance (ANOVA), followed by *post-hoc* Tukey test. The data using postmortem brain samples were analyzed using Mann-Whitney U-test. Correlation was determined by Pearson correlation. The *P*-values of less than 0.05 were considered statistically significant.

## Results

### Prophylactic effects of (*R*)-ketamine on depression-like phenotype, splenomegaly, and systemic inflammation after LPS administration

Saline or LPS (1.0 mg/kg) was administered to mice 6 days after injection of saline or (*R*)-ketamine (10 mg/kg) (Fig. 1A). Body weight of mice was significantly decreased 24 h after LPS injection (Fig. 1B). Pretreatment with (*R*)-ketamine significantly attenuated LPS-induced body weight loss (Fig. 1B). There were no significant changes in the locomotor activity among the three groups (Fig. 1C). Pretreatment with (*R*)-ketamine significantly ameliorated LPS-induced increase in the immobility time of FST (Fig. 1D). In contrast, pretreatment with (*R*)-norketamine (10 mg/kg) or (2*R*,6*R*)-HNK (10 mg/kg), two metabolites of (*R*)-ketamine, did not show prophylactic effects for body weight loss, splenomegaly and depression-like phenotype in LPS-treated mice (Figure S1).

We previously reported that LPS caused the splenomegaly and the increased ratio of spleen weight to body weight in the mice, and that spleen weight was associated with systemic inflammation [50]. Pretreatment with (*R*)-ketamine (10 mg/kg) significantly attenuated the splenomegaly and the increased ratio of spleen weight to body weight in mice after LPS administration (Fig. 1E, F). Pretreatment with (*R*)-ketamine significantly attenuated the increased blood levels of IL-6 and TNF-α in the LPS-treated mice (Fig. 1G, H). There were significantly positive correlations between plasma IL-6 (or TNF-α) levels and spleen weight in the three groups (Fig. 1I, J). The data suggest that LPS-induced systemic inflammation might be related with spleen weight, consistent with our previous reports [50, 62].

### RNA-sequencing analysis of PFC samples

**Fig. 2:**
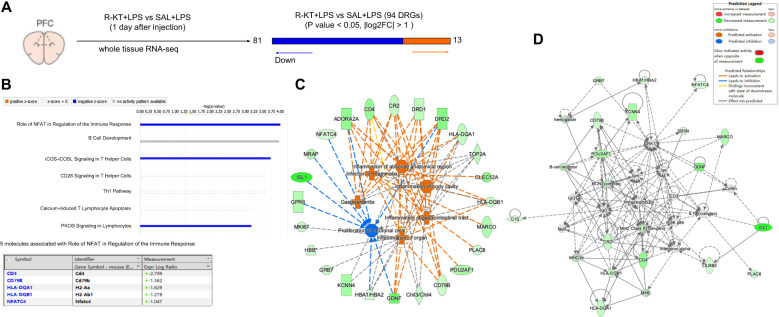
Canonical pathway, protein functional network and diseases analysis for differentially genes in the PFC after LPS injection. **A** Adult mice were i.p. injected with LPS (1.0 mg/kg) 6 days after i.p. administration of (*R*)-ketamine (10 mg/kg) or saline (10 ml/kg). For RNA-sequencing, PFC samples were collected 24 h after injection of LPS. **B** Top 7 canonical pathways altered in the PFC were identified by Ingenuity Pathway Analysis (IPA). The role of NFAT in regulation of the immune response signaling pathway had the highest inhibition scores (*P* = 9.85E−05, z-score = –2.236). **C** IPA constructs identified relationships between the differentially expressed genes and associated disorders. **D** The most significant molecular network by IPA pathway enrichment analysis. IPA Z-score indicates whether the pathway is predicated to be inhibited (blue) or activated (red). In some cases, activation or inhibition cannot be predicated (gray). The shapes of the proteins imply their molecular classes as outlined in the legend. Solid lines indicate direct interaction whereas dashed line correspond to indirect relationship among the interacting proteins. The arrows indicate modulatory effect of a protein on its interacting proteins.

To identify the novel molecular targets for the prophylactic effects of (*R*)-ketamine (10 mg/kg, 6 days before), we collected PFC samples 24 h after administration of LPS (1.0 mg/kg). We performed RNA-sequencing analysis of PFC samples from animals treated with either (*R*)-ketamine or saline (Fig. 2A). The canonical pathway results identified a total of 7 pathways. Among these pathways, the role of NFAT (nuclear factor of activated T cells) in regulation of immune response signaling pathway had the highest inhibition score, and 5 genes including *CD4*, *CD79b*, *H2-ab1*, *H2-aa*, *Nfatc4* are related to NFAT pathway (Fig. 2B). Subsequent diseases and functions analysis shows that the differentially expressed genes were associated with inflammation (Fig. 2C). In the network analysis, we observed 18 genes, and the top functions of this network included cell morphology, cell-to-cell signaling interaction, and immunoglobulin (Fig. 2D).

**Fig. 3:**
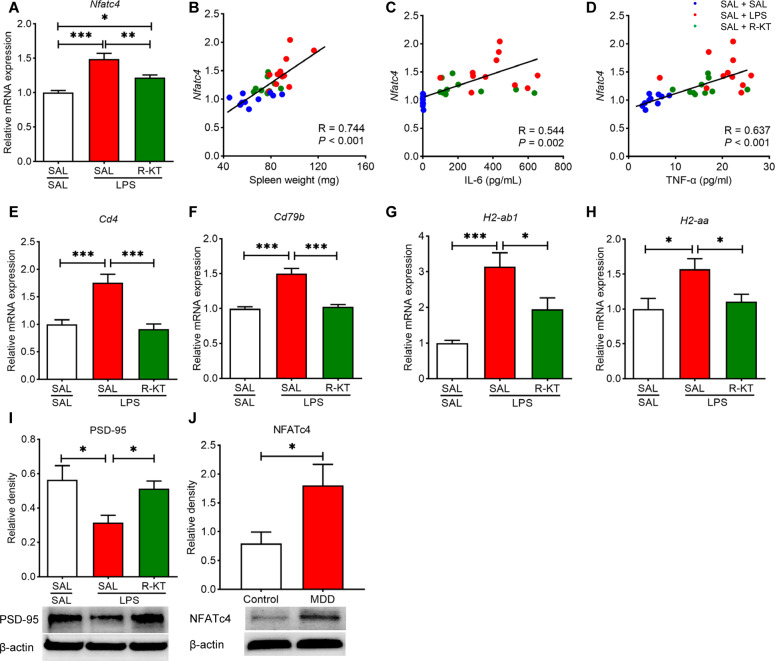
Prophylactic effects of (*R*)-ketamine on the expression of NFATc4 in the PFC after LPS injection. **A** *Nfatc4* mRNA in the PFC (one way ANOVA: F_2,28_ = 17.84, *P* < 0.0001). **B** There was a positive correlation (*R* = 0.744, *P* < 0.0001) between spleen weight and *Nfatc4* mRNA in the PFC. **C** There was a positive correlation (*R* = 0.544, *P* = 0.002) between plasma interleukin (IL)-6 and *Nfatc4* mRNA in the PFC. **D** There was a positive correlation (*R* = 0.637, *P* < 0.0001) between plasma tumor necrosis factor (TNF)-α and *Nfatc4* mRNA in the PFC. **E** *Cd4* mRNA in the PFC (one way ANOVA: F_2,28_ = 16.16, *P* < 0.0001). **F** *Cd79b* mRNA in the PFC (one way ANOVA: F_2,28_ = 30.40, *P* < 0.0001). **G** *H2-ab1* mRNA in the PFC (one way ANOVA: F_2,28_ = 12.81, *P* = 0.0001). **H** *H2-aa* mRNA in the PFC (one way ANOVA: F_2,28_ = 5.617, *P* = 0.009). **I** The protein expression of PSD-95 in the PFC (one way ANOVA: F_2,27_ = 5.506, *P* = 0.010). The data represent mean ± S.E.M. (*n* = 9–11). ^*^P < 0.05, ^**^P < 0.01, ^***^P < 0.001. **J** The protein expression of NFATc4 in the parietal cortex from controls (*n* = 15) and MDD patients (*n* = 15) (Man–Whitney U-test: U = 60.00, *P* = 0.030). The data represent mean ± S.E.M. (*n* = 15). ^*^P < 0.05.

Next, we measured gene expression of several genes (*Nfatc4, Cd4, Cd79b, H2-ab1*, and *H2-aa*) for NFATc4 signaling in the PFC samples. We found increased expression of *Nfatc4, Cd4, Cd79b, H2-Ab1*, and *H2-Aa* in the PFC from LPS-treated mice (Fig. 3A, E–H). Pretreatment with (*R*)-ketamine (10 mg/kg) significantly attenuated the increased expression of these genes in the PFC of LPS-treated mice (Fig. 3A, E–H). There were positive correlations between expression of *Nfatc4* in the PFC and spleen weight (or blood levels of IL-6, TNF-α) from three groups (Fig. 3B, C), indicating that *Nfatc4* expression in the PFC may be associated with systemic inflammation.

Western blot analysis showed that pretreatment with (*R*)-ketamine (10 mg/kg) significantly ameliorated the reduction of postsynaptic density protein 95 (PSD-95) in the PFC of LPS-treated mice (Fig. 3I). Furthermore, Western blot analysis using postmortem brain samples showed that the levels of NFATc4 in the parietal cortex from MDD patients were significantly higher than those of controls (Fig. 3J).

Pretreatment with (*R*)-ketamine (10 mg/kg) significantly attenuated splenomegaly and increased levels of IL-6 and TNF-α in the LPS-treated mice (Figure S2). Furthermore, there were positive correlations between spleen weight (or IL-6, TNF-α) and expression of several genes (i.e., *Cd4, Cd79b, H2-Ab1*) in the PFC from three groups (Figure S2). The data suggest that gene expression of NFATc4 signaling in the PFC may be associated with systemic inflammation.

### Effects of NFAT inhibitors on LPS-induced depression-like phenotype

**Fig. 4:**
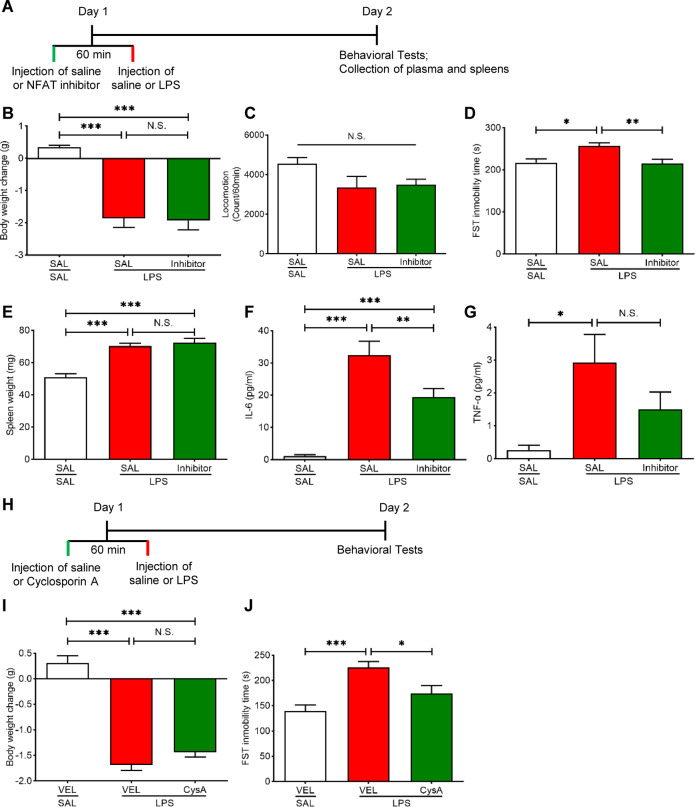
Effects of NFAT inhibitors on depression-like phenotype, spleen weight and pro-inflammatory cytokines after LPS injection. **A** Treatment schedule. Mice were i.p. injected with LPS (1.0 mg/kg) or saline (10 ml/kg). NFAT inhibitor (10 μM, 2 μl) or saline (2 μl) was administered i.c.v. to mice 60 min prior to LPS injection. Locomotion test and forced swimming test (FST) were performed 23 and 24 h after the injection of saline or LPS, respectively. Blood and spleens were collected after behavioral tests. **B** Body weight change (one way ANOVA: F_2,29_ = 27.72, *P* < 0.0001). **C** Locomotion test (one way ANOVA: F_2,29_ = 2.589, *P* = 0.092). **D** FST (one way ANOVA: F_2,29_ = 6.486, *P* = 0.005). **E** Spleen weight (one way ANOVA: F_2,28_ = 26.41, *P* < 0.0001). **F** Plasma levels of IL-6 (one way ANOVA: F_2,28_ = 28.67, *P* < 0.0001). **G** Plasma levels of TNF-α (one way ANOVA: F_2,28_ = 5.013, *P* = 0.014). **H** Treatment schedule. Mice were i.p. injected with LPS (1.0 mg/kg) or saline (10 ml/kg). Cyclosporin A (CysA: 40 mg/kg) or vehicle (10% DMSO, 10 ml/kg) was i.p. injected to mice 60 min prior to LPS injection. Locomotion test and FST were performed 23 and 24 h after the injection of saline or LPS, respectively. **I** Body weight change (one way ANOVA: F_2,27_ = 90.99, *P* < 0.0001). **J** FST (one way ANOVA: F_2,27_ = 10.86, *P* = 0.0003). The data represent mean ± S.E.M. (*n* = 10–12). ^*^P < 0.05, ^**^P < 0.01, ^***^P < 0.001. N.S., not significant.

To study the role of NFATc4 signaling in LPS-induced depression-like phenotype, two NFAT inhibitors (NFAT inhibitor and CysA) were used (Fig. 4A, H). Pretreatment with the NFAT inhibitor (10 μM, 2 μl, i.c.v., 60 min) significantly attenuated LPS-induced increase in the immobility time of FST (Fig. 4D), without significant effects on LPS-induced body weight loss, splenomegaly, and locomotion (Fig. 4B–E). The NFAT inhibitor significantly attenuated increased blood levels of IL-6 in the LPS-treated mice (Fig. 4F). The NFAT inhibitor slightly attenuated increased blood levels of TNF-α in the LPS-treated mice although statistical analysis did not reach significance (Fig. 4G).

Furthermore, pretreatment with NFAT inhibitor CysA (40 mg/kg, i.p., 60 min) significantly inhibited LPS-induced increase in the immobility time of FST (Fig. 4J) without significant effects in the body weight loss (Fig. 4I). These data show that similar to (*R*)-ketamine, NFAT inhibitors can show prophylactic effects for LPS-induced depression-like behavior.

### Effects of NFATc4 knockdown on LPS-induced depression-like phenotype

**Fig. 5:**
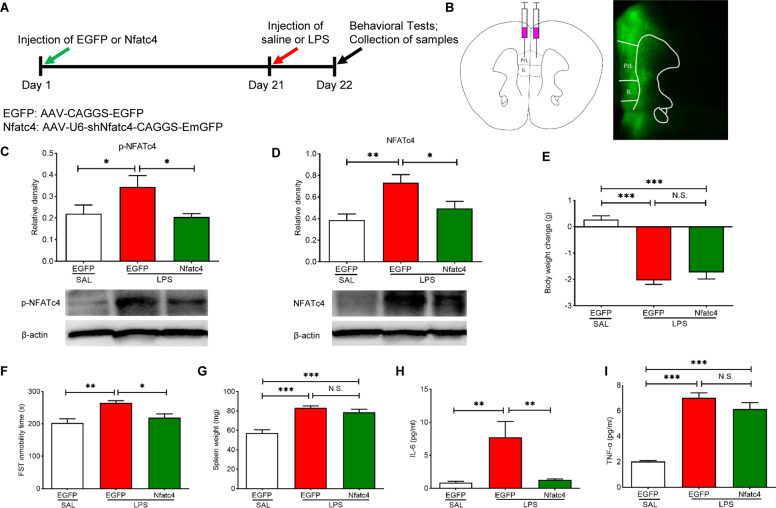
Effects of NFATc4 knockdown on depression-like phenotype, spleen weight and inflammatory cytokines after LPS injection. **A** Treatment schedule. AAV-U6-shNfatc4-CAGGS-EmGFP or AAV-CAGGS-EGFP was injected bilaterally to mPFC 21 days prior to saline (10 ml/kg) or LPS (1.0 mg/kg) injection. Locomotion test and FST were performed 23 and 24 h after the injection of saline or LPS, respectively. Blood, spleen and mPFC were collected after behavioral tests. **B** Schematic of AAV-mediated *Nfatc4* down expression in the mPFC. The diagram shows the AAV constructs and stereotaxic injection of AAV into the mPFC. **C** The protein expression of phosphorylated NFATc4 (p-NFATc4) in the mPFC (one way ANOVA: F_2,26_ = 5.440, *P* = 0.011). **D** The protein expression of total NFATc4 in the mPFC (one way ANOVA: F_2,26_ = 6.673, *P* = 0.005). **E** Body weight change (one way ANOVA: F_2,26_ = 36.65, *P* < 0.0001). **F** FST (one way ANOVA: F_2,26_ = 8.409, *P* = 0.002). **G** Spleen weight (one way ANOVA: F_2,26_ = 23.94, *P* < 0.0001). **H** Plasma levels of IL-6 (one way ANOVA: F_2,26_ = 8.580, *P* = 0.001). **I** Plasma levels of TNF-α (one way ANOVA: F_2,26_ = 79.13, *P* < 0.0001). The data represent mean ± S.E.M. (n = 9–11). ^*^P < 0.05, ^**^P < 0.01, ^***^P < 0.001. N.S., not significant.

We further studied the impact of NFATc4 knockdown on LPS-induced depression-like phenotype, splenomegaly, and increased plasma inflammatory cytokines. AAV-U6-shNfatc4-CAGGS-EmGFP or AAV-CAGGS-EGFP were stereotaxic injected into the mPFC to induce knockdown of NFATc4 in the mPFC (Fig. 5A). Western blotting analysis confirmed the knockdown efficiency of NFATC4 and p-NFATC4 in the mPFC (Fig. 5C, D). NFATc4 knockdown significantly ameliorated LPS-induced increase in the immobility time of FST (Fig. 5F). In contrast, NFATc4 knockdown in the mPFC did not affect body weight loss (Fig. 5E) and splenomegaly in the LPS-treated mice (Fig. 5G). NFATc4 knockdown in the mPFC significantly attenuated increased levels of IL-6, but not TNF-α, in the LPS-treated mice (Fig. 5H, I). The data suggest that NFATc4 in the mPFC plays a role in depression-like phenotype and increases in blood levels of IL-6 of LPS-treated mice.

### The roles of TrkB in the prophylactic effects of (*R*)-ketamine in LPS model

We previously reported the role of brain-derived neurotrophic factor (BDNF) and its receptor, tropomyosin-receptor-kinase B (TrkB) signaling in the beneficial actions of (*R*)-ketamine [32, 37, 53–56]. To investigate the roles of BDNF-TrkB signaling in the prophylactic effects of (*R*)-ketamine on LPS-induced depression-like phenotype, TrkB antagonist ANA-12 (0.5 mg/kg) was injected to mice 30 min before injection of (*R*)-ketamine (Figure S3). There were no changes in locomotion among the five groups (Figure S3B). Pretreatment with ANA-12 significantly blocked the antidepressant-like effects of (*R*)-ketamine in the LPS-treated mice (Figure S3C). Our data suggest that (*R*)-ketamine shows prophylactic effects on LPS-induced depression-like phenotype via BDNF-TrkB signaling.

## Discussion

The main findings of this study are as follows: First, pretreatment (6 days) with (*R*)-ketamine could ameliorate LPS-induced depression-like phenotype, splenomegaly, and increased blood levels of pro-inflammatory cytokines in mice. In contrast, (*R*)-norketamine and (2*R*,6*R*)-HNK did not show prophylactic effects in the same model. Second, RNA-sequencing and IPA revealed the role of NFATc4 signaling in the PFC for prophylactic effects of (*R*)-ketamine in the LPS-induced model. RT-PCR revealed the increased expression of several genes (*Nfatc4, Cd4, Cd79b, H2-Ab1*, *H2-Aa*) of NFATc4 signaling in the PFC of LPS-treated mice. Furthermore, (*R*)-ketamine significantly attenuated the increased expression of these genes in the PFC of LPS-treated mice. There were positive correlations between the expression of *Nfatc4* mRNA in the PFC and spleen weight (or blood levels of IL-6, TNF-α) from three groups. In addition, we found the increased expression of NFATC4 protein in the parietal cortex of MDD patients compared to controls. Third, pharmacological inhibitors of NFAT showed prophylactic antidepressant-like effects in the LPS-treated mice, indicating a role of NFAT signaling in the prophylactic antidepressant-like effects of (*R*)-ketamine. Fourth, knockdown of *Nfatc4* gene in the mPFC by AAV blocked LPS-induced increases in the immobility time of FST, suggesting a role of NFATc4 in the mPFC in the prophylactic effects of (*R*)-ketamine. Lastly, pretreatment with TrkB inhibitor ANA-12 significantly blocked prophylactic effects of (*R*)-ketamine in the LPS-treated mice. Overall, it appears likely that (*R*)-ketamine can exert sustained prophylactic antidepressant-like effects by decreasing NFATc4 signaling in the PFC.

We found that (*R*)-ketamine showed a sustained (6 days) prophylactic effect in inflammation model of depression; however, (*R*)-norketamine and (2*R*,6*R*)-HNK did not show prophylactic effects in the same model. We previously reported that (*R*)-norketamine and (2*R*,6*R*)-HNK did not show antidepressant-like effects in LPS-induced inflammation, LH, and CSDS models of depression [35, 63]. Therefore, it is likely that (*R*)-ketamine itself, but not these metabolites, could have prophylactic effects in LPS-treated mice.

Despite of short half-life of (*R*)-ketamine in rodents [33, 34], (*R*)-ketamine showed sustained (6 days) prophylactic effects in LPS-treated mice. The data suggest that altered signaling pathway induced by (*R*)-ketamine may play a role in its prophylactic effects. RNA-seq analysis and IPA identified a role of NFATc4 signaling in the PFC for prophylactic effects of (*R*)-ketamine. Using two NFAT inhibitors and AAV for *Nfatc4*, we found that NFATc4 signaling in the mPFC might play a role in the sustained prophylactic effects of (*R*)-ketamine for LPS-induced depression.

The transcription factor NFATc4 is localized in neuron, but not astrocyte, microglia, and oligodendrocyte, in the brain [64]. Interestingly, NFATc4 is demonstrated to play a key role in BDNF-mediated synaptic plasticity, resulting in long-term changes in neuronal functions [52, 65]. In this study, we found positive correlations between *Nfatc4* gene expression in the PFC and spleen weight (or pro-inflammatory cytokines). Considering the crucial role of NFATc4 in immune system [66, 67], it seems that NFATc4 in the PFC may regulate systemic inflammation in mice via brain-body crosstalk. However, the precise mechanisms underlying (*R*)-ketamine-induced reduction of NFATc4 signaling are currently unknown.

We previously reported that LPS caused splenomegaly in mice, and that spleen weight of LPS-treated mice was associated with blood levels of pro-inflammatory cytokines in these mice [50, 62]. In this study, pretreatment with (*R*)-ketamine ameliorated splenomegaly in the LPS-treated mice through anti-inflammatory effects although (*R*)-ketamine was washout from the body. In contrast, the NFAT inhibitors or AAV in the mPFC did not affect splenomegaly in the LPS-treated mice. It is unlikely that gene knockdown of *NFATc4* by AAV in the mPFC can affect LPS-induced splenomegaly in mice. Thus, it seems that other mechanisms except NFATc4 may play a role in the effects of (*R*)-ketamine on the LPS-induced splenomegaly. In contrast, gene knockdown of *NFATc4* by AAV in the mPFC significantly attenuated increased blood levels of IL-6 in the LPS-treated mice via brain-body communication. Precise mechanisms underlying the relationship between gene knockdown of *Nfatc4* in the mPFC and blood levels of IL-6 remain unclear. Recently, we reported that splenomegaly in CSDS susceptible mice could be normalized after single injection of (*R*)-ketamine [68]. It is possible that brain–spleen axis may play a role in the beneficial effects of (*R*)-ketamine on depression-like phenotype and splenomegaly [19, 22], although further study is needed.

Depression has high rate of recurrence, resulting in significant personal and public health consequences [69]. Therefore, prevention of recurrence using cognitive behavioral therapy and pharmacological treatment is extremely important. Given potent prophylactic effects of (*R*)-ketamine, it is possible that (*R*)-ketamine might prevent the recurrence in depressed patients. It is, therefore, of interest to investigate whether (*R*)-ketamine can reduce the recurrent rate in depressed patients.

In conclusion, this study shows that NFATc4 signaling in the PFC might contribute to the prophylactic effects of (*R*)-ketamine in inflammation model of depression. It is likely that (*R*)-ketamine or NFATc4 inhibitors may produce prophylactic effects for inflammation-related depression in humans.

## Supplementary information

Supplemental information

## Data Availability

The RNA sequencing data have been deposited to the NCBI Sequence Read Archive and are available at the accession number PRJNA768662.
