# Supplementary material for: Nuclear factor of activated T cells 4 in the prefrontal cortex is required for prophylactic actions of (R)-ketamine
Source: Transl Psychiatry. 2022 Jan 21;12:27. doi: 10.1038/s41398-022-01803-6 (PMC8782904; doi:10.1038/s41398-022-01803-6)
Supplement: Supplementary file 1 — Supplemental information [file 41398_2022_1803_MOESM1_ESM.docx]

**Supplemental Information**

**Nuclear factor of activated T cells 4 in the medial prefrontal cortex is required for prophylactic actions of (*R*)-ketamine**

Li Ma^1,2^, Jiancheng Zhang^1^, Yuko Fujita^1^, Youge Qu^1^, Jiajing Shan^1^, Xiayun Wan^1^, Xingming Wang^1^, Tamaki Ishima^1^, Kenta Kobayashi^3^, Long Wang^2^ and Kenji Hashimoto^1^

^1^Division of Clinical Neuroscience (LM, JZ, YF, YQ, JS, XW, XW, TI, KH), Chiba University Center for Forensic Mental Health, Chiba 260-8670, Japan, ^2^Department of Anesthesiology (LM, LW), Renmin Hospital of Wuhan University, Wuhan 430060, Hubei Province, China, and ^3^Section of Viral Vector Development (KK), Center for Genetic Analysis of Behavior, National Institute for Physiological Sciences, Okazaki, Aichi 444-8585, Japan.

**Correspondence:** Dr. Kenji Hashimoto. E-mail: hashimoto@faculty.chiba-u.jp.

**Methods**

**Behavioral tests**

Behavioral tests such as locomotion test and forced swimming test (FST) were performed in a blind manner.

Locomotion test: Mice were placed in experimental cages (L560 × W560 × H330 mm), and locomotor activity was counted by the SCANET MV-40 (MELQUEST, Toyama, Japan). The cumulative exercise was recorded for 60 min. All cages were cleaned between testing session. FST: Animals were tested in an automated forced-swim apparatus using SCANET MV-40 (MELQUEST Co., Ltd., Toyama, Japan). The mice were placed individually in a cylinder (Diameter 23 cm; Height 21 cm), containing 15 cm of 23 ± 1℃ warm water. Immobility time was calculated by subtracting active time from total time, using the apparatus analysis software. Cumulative immobility time was scored for 6 min during the test.

**Western Blotting**

Human postmortem parietal cortex (Brodmann area 7: BA7) from normal controls (n =15) and patients with major depressive disorder (MDD) (n =15) were obtained from the Stanley Foundation Brain Collection (Bethesda, MD, USA). The demographic, clinical, and storage information for cases has been previously published [[1](https://www.ncbi.nlm.nih.gov/pmc/articles/PMC7243919/#R17)]. Each group was matched according to several parameters, including age at death, gender, postmortem interval (PMI), brain pH, and brain weight [2-5]. Human brain samples were stored at −80°C until biochemical analyses.

Tissue samples were homogenized in ice-cold Laemmli lysis buffer and then centrifuged at 3,000 g for 10 min at 4°C to get the supernatants. The protein concentrations were quantified using a bicinchoninic acid (BCA) protein assay kit (Bio-Rad, Hercules, CA). The samples were then mixed with an equal volume of loading buffer (125 mM Tris/HCl, pH 6.8, 20% glycerol, 0.1% bromophenol blue, 10% β-mercaptoethanol, and 4% sodium dodecyl sulfate) and boiled for 10 min at 95°C. Proteins were separated using 10% sodium dodecyl sulfate–polyacrylamide gel electrophoresis (SDS-PAGE) gels (Mini-PROTEAN^®^ TGX™ Precast Gel; Bio-Rad) and then transferred onto polyvinylidene difluoride (PVDF) membranes using a Trans Blot Mini Cell (Bio-Rad). The membranes were blocked with 5% skim milk in TBS with 0.1% Tween 20 (TBST) for 1 h at room temperature and then incubated with the following primary antibodies: against PSD-95 (1:1000, Cat No.: 51-6900, Invitrogen, Camarillo, CA, USA), p-NFAc4 (1:1000, Cat No.: SAB4503946, Sigma-Aldrich Co., Ltd., St Louis, MO, USA), NFATc4 (1:1000, Cat No.:AV32040, Sigma-Aldrich Co., Ltd., St Louis, MO, USA), β-actin (1:10,000, Sigma-Aldrich Co., Ltd., St Louis, MO, USA) overnight at 4°C. After three washes with TBST, the membranes were incubated with horseradish peroxidase (HRP)-conjugated anti-rabbit or anti-mouse antibody (1:5,000) for 1 h at room temperature. After being washed three washes with TBST, the bands were visualized using enhanced chemiluminescence (ECL) plus the Western Blot Detection system (GE Healthcare Bioscience) and captured by ChemiDoc™ Touch Imaging System (170–01,401; Bio-Rad Laboratories, Hercules, CA). The images were subjected to grayscale analysis using Image Lab™ 3.0 software (Bio-Rad Laboratories).

**References**

1. Torrey EF, Webster M, Knable M, Johnston N, Yolken RH. The Stanley foundation brain collection and neuropathology consortium. Schizophr. Res. 2000;44:151–155.
2. Hashimoto K, Sawa A, Iyo M. Increased levels of glutamate in brains from patients with mood disorders. Biol. Psychiatry 2007;62:1310–1316.
3. Yang B, Ren Q, Zhang JC, Chen QX, Hashimoto K. Altered expression of BDNF, BDNF pro-peptide and their precursor proBDNF in brain and liver tissues from psychiatric disorders: rethinking the brain-liver axis. Transl. Psychiatry 2017;7:e1128.
4. Ren Q, Ma M, Ishima T, Morisseau C, Yang J, Wagner KM, [et](http://www.ncbi.nlm.nih.gov/pubmed/?term=Krystal%20JH%5BAuthor%5D&cauthor=true&cauthor_uid=10686270) al. Gene deficiency and pharmacological inhibition of soluble epoxide hydrolase confers resilience to repeated social defeat stress. Proc. Natl. Acad. Sci. USA 2016;113:E1944–952.
5. Zhang JC, Yao W, Dong C, Han M, Shirayama Y, Hashimoto K. Keap1-Nrf2 signaling pathway confers resilience versus susceptibility to inescapable electric stress. Eur. Arch. Psychiatry Clin. Neurosci. 2018;268:865–870.


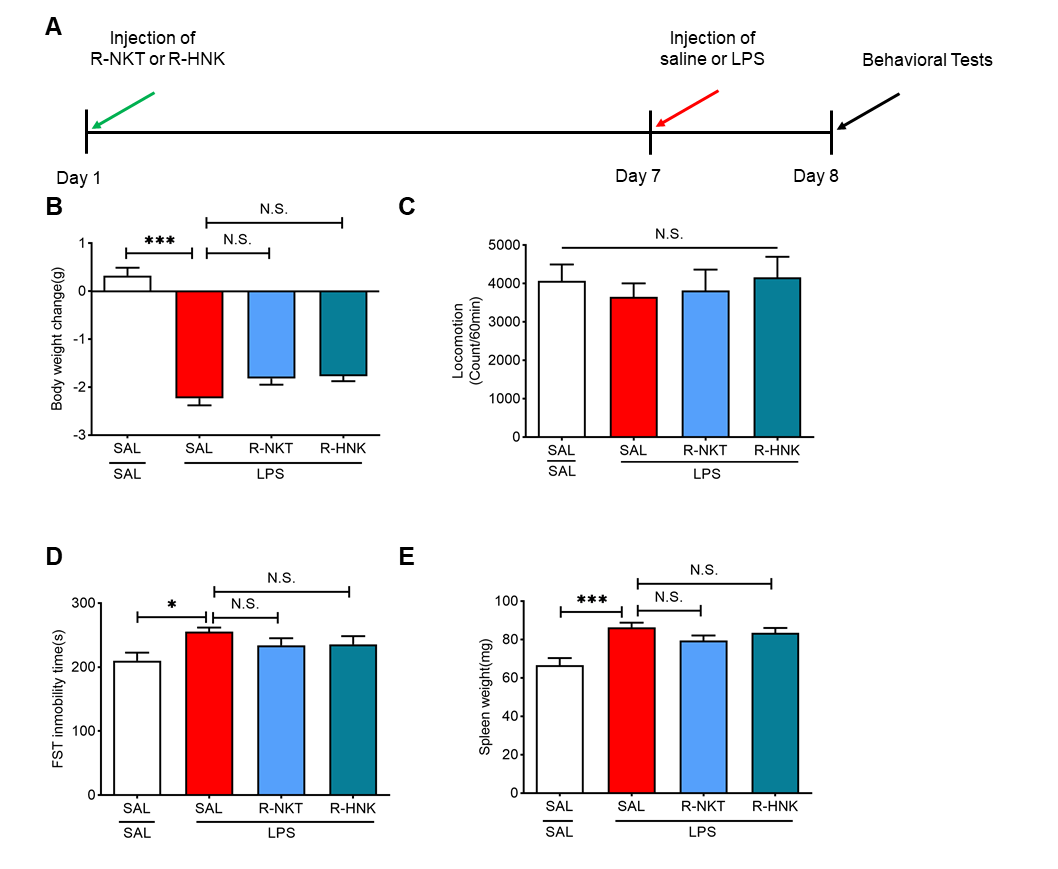
 **Figure S1. (*R*)-ketamine metabolites had no prophylactic effects on depression-like phenotype and spleen weight after LPS injection**.

(**A**): Treatment schedule. Adult mice were i.p. injected with lipopolysaccharides (LPS, 1.0 mg/kg) or saline (10 ml/kg). (*R*)-norketamine (R-NKT; 10 mg/kg), (2*R*,6*R*)-hydroxynorketamine (R-HNK; 10 mg/kg) or saline (10 ml/kg) was i.p. injected to mice 6 days before saline or LPS injection. Locomotion test and forced swimming test (FST) were performed 23 and 24 h after the injection of saline or LPS, respectively. Spleens were collected after behavioral tests. (**B**): Body weight change (one way ANOVA: F_3,35_ = 72.09, *P* < 0.0001). (**C**): Locomotion test (one way ANOVA: F_3,35_ = 0.263, *P* = 0.852). (**D**): FST (one way ANOVA: F_3,35_ = 3.338, *P* = 0.030). (**E**): Spleen weight (one way ANOVA: F_3,35_ = 9.076, *P* = 0.0001). The data represent mean ± S.E.M. (n = 8–12). ^*^*P* < 0.05, ^**^*P* < 0.01, ^***^*P* < 0.001. N.S., not significant.


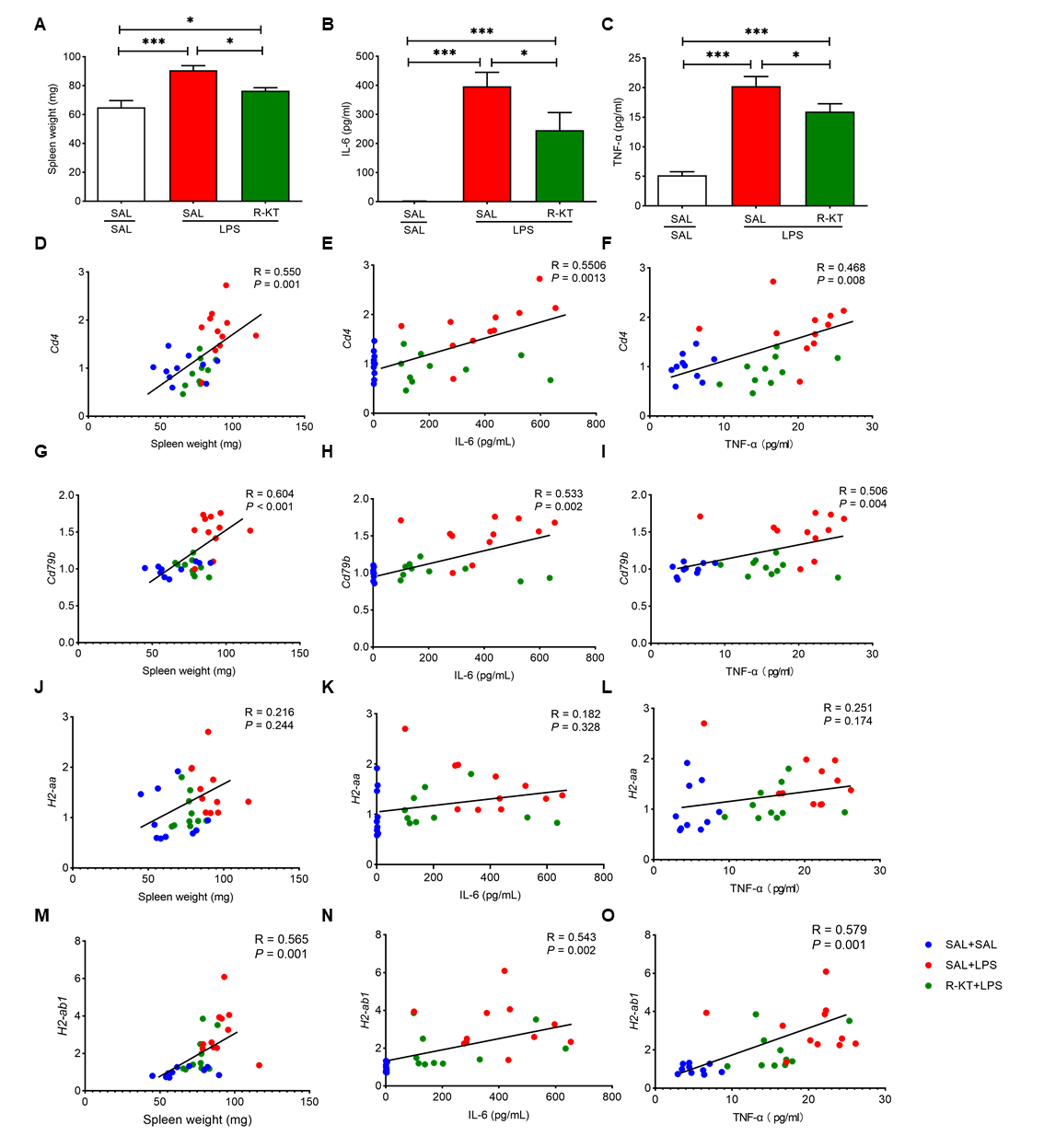
**Figure S2. Correlation analysis of *Nfatc4*-related subfamily with spleen weight and plasma pro-inflammatory cytokines**.

(**A**): Spleen weight (one way ANOVA: F_2,28_ = 14.46, *P* < 0.0001). (**B**): Plasma levels of interleukin (IL)-6 (one way ANOVA: F_2,28_ = 22.63, *P* < 0.0001). (**C**): Plasma levels of tumor necrosis factor (TNF)-α (one way ANOVA: F_2,28_ = 43.26, *P* < 0.0001). The data represent mean ± S.E.M. (n = 10–11). ^*^*P* < 0.05, ^**^*P* < 0.01, ^***^*P* < 0.0001. **(D-F):** There was a positive correlation between *Cd4* mRNA and spleen weight (**D**; R = 0.550, *P* = 0.001), plasma IL-6 (**E**; R = 0.551, *P* = 0.001), or plasma TNF-α (**F**; R = 0.468, *P* = 0.008). **(G-I)**: There were positive correlations between spleen weight and *Cd79b* mRNA (**G**; R = 0.604, *P =* 0.0003), plasma IL-6 (**H**; R = 0.533, *P* = 0.002), or plasma TNF-α (**I**; R = 0.505, *P* = 0.004) was observed. **(J-L)**: There were no correlations between *H2-aa* mRNA and spleen weight (**J**; R = 0.216, *P* = 0.244), plasma IL-6 (**K**; R = 0.182, *P* = 0.328), or plasma TNF-α (**L**; R = 0.251, *P* = 0.174). **(M-O):** There were positive correlations between *H2-ab1* mRNA and spleen weight (**M**; R = 0.566, *P* = 0.001), plasma IL-6 (**N**; R = 0.543, *P* = 0.002), or plasma TNF-α (**O**; R = 0.579, *P* = 0.001).


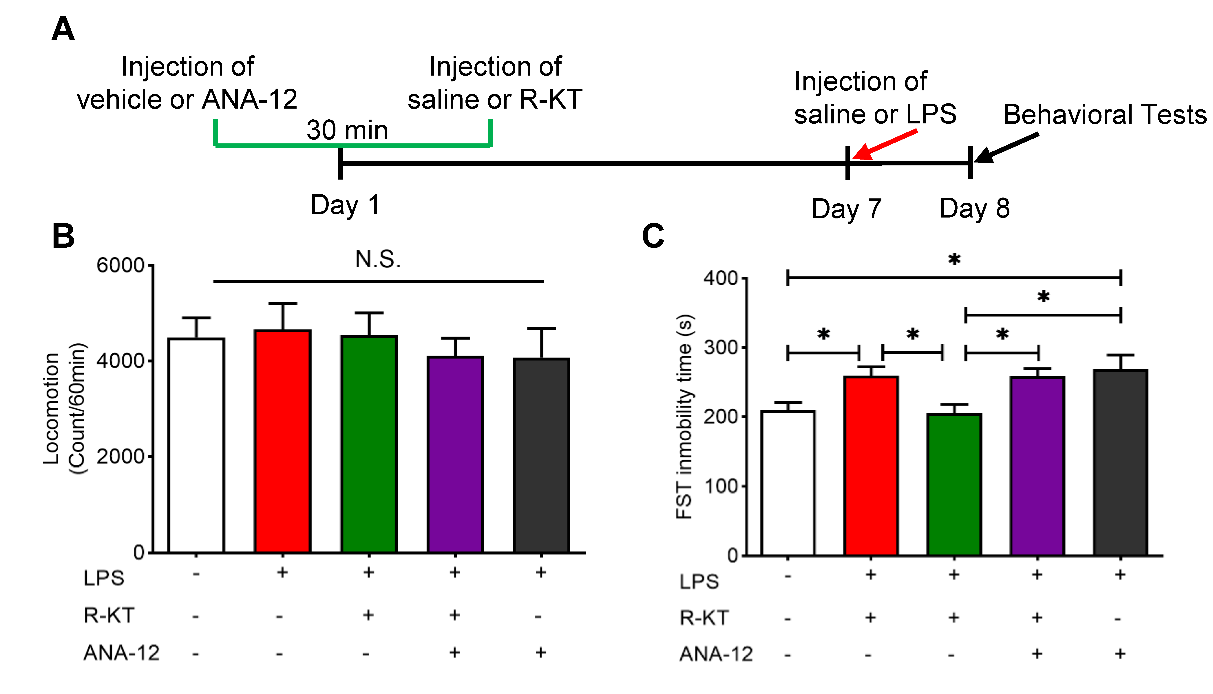


**Figure S3. Effects of TrkB antagonist ANA-12 on prophylactic protective effects of (*R*)-ketamine on LPS-induced depression-like phenotype** (**A**): Treatment schedule. (*R*)-ketamine (10 mg/kg) or saline (10 ml/kg) was i.p. injected to mice 6 days before injection of saline (10 ml/kg) or LPS (1.0 mg/kg). Locomotion test and FST were performed 23 and 24 h after the injection of saline or LPS, respectively. ANA-12 (0.5 mg/kg) or vehicle (17% DMSO) was i.p. injected to mice 30 minutes before i.p. administration of saline or (*R*)-ketamine. (**B**): Locomotion test (one way ANOVA: F_4,82_ =0.290, *P* = 0.883). (**C**): FST (one way ANOVA: F_4,82_ =5.413, *P* =0.001). The data represent mean ± S.E.M. (n =10-20). ^*^*P* < 0.05. N.S., not significant.
